# Epigenetic Studies of Chinese Herbal Medicine: Pleiotropic Role of DNA Methylation

**DOI:** 10.3389/fphar.2021.790321

**Published:** 2021-12-07

**Authors:** Wenqian Guo, Han Ma, Chong-Zhi Wang, Jin-Yi Wan, Haiqiang Yao, Chun-Su Yuan

**Affiliations:** ^1^ School of Traditional Chinese Medicine, Beijing University of Chinese Medicine, Beijing, China; ^2^ National Institute of TCM Constitution and Preventive Medicine, Beijing University of Chinese Medicine, Beijing, China; ^3^ Tang Center for Herbal Medicine Research, The University of Chicago, Chicago, IL, United States; ^4^ Department of Anesthesia and Critical Care, The University of Chicago, Chicago, IL, United States

**Keywords:** epigenetics, DNA methylation, Chinese herbal medicine, constitution theory, ethnopharmacology

## Abstract

Accumulating knowledge has been achieved on DNA methylation participating in numerous cellular processes and multiple human diseases; however, few studies have addressed the pleiotropic role of DNA methylation in Chinese herbal medicine (CHM). CHM has been used worldwide for the prevention and treatment of multiple diseases. Newly developed epigenetic techniques have brought great opportunities for the development of CHM. In this review, we summarize the DNA methylation studies and portray the pleiotropic role of DNA methylation in CHM. DNA methylation serves as a mediator participating in plant responses to environmental factors, and thus affecting CHM medicinal plants growth and bioactive compound biosynthesis which are vital for therapeutic effects. Furthermore, DNA methylation helps to uncover the pharmaceutical mechanisms of CHM formulae, herbs, and herbal-derived compounds. It also provides scientific validation for constitution theory and other essential issues of CHM. This newly developed field of DNA methylation is up-and-coming to address many complicated scientific questions of CHM; it thus not only promotes disease treatment but also facilitates health maintenance.

## Introduction

Chinese herbal medicine (CHM), originated in ancient China, has been used worldwide to prevent and treat different medical conditions (Ang-Lee et al., 2001; Pang et al., 2017). With multi-target and holistic approaches, CHMs have shown advantages in managing multi-factorial, systemic, and complex diseases, such as cancer, metabolic syndrome, and autoimmune diseases (Zhong et al., 2016). Newly developed biomedical technologies, such as “-omics” analysis, have triggered the transformation of the scientific paradigm and shifted from reductionism to holism (Kellogg et al., 2018; Lippolis et al., 2018). These new approaches have echoed the philosophy and therapeutics of CHM (Fardet and Rock, 2014; Delker and Mann, 2017; Hu and Su, 2017).

**FIGURE 1:**
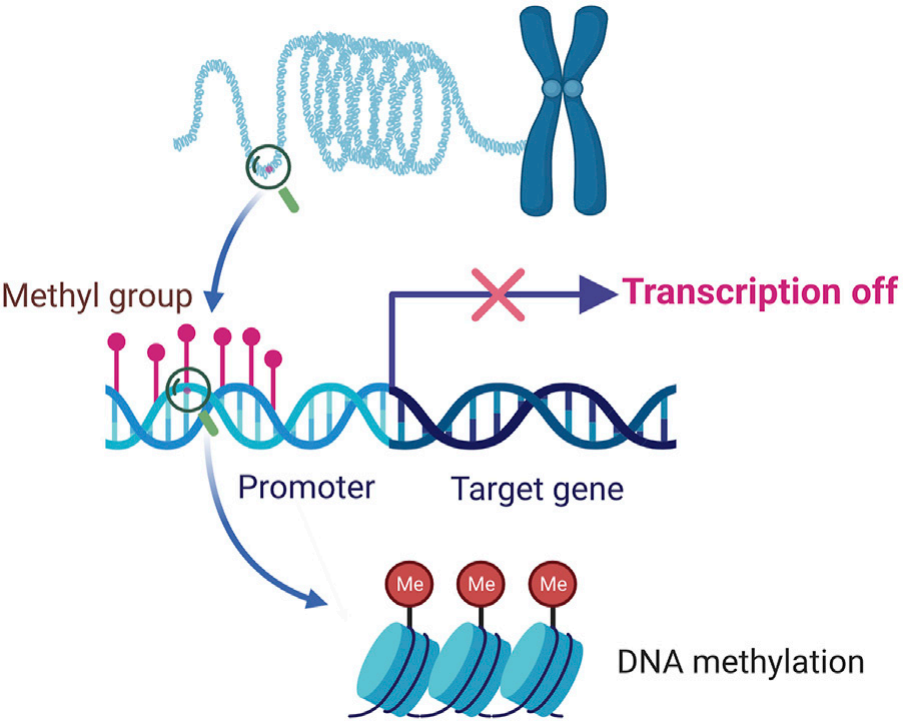
The primary function of DNA methylation. As one of the most important epigenetic mechanisms, DNA methylation refers to the process of methyl groups being added to the DNA molecule. When DNA methylation occurs in promoter regions, the gene transcription may be repressed without changing the DNA sequence.

The neologism “-omics,” including genomics, proteomics, and metabolomics, refers to the collective characterization and quantification of pools of biological molecules that translate into the structure, function, and dynamics of an organism (Kedaigle and Fraenkel, 2018). The current “-omics” studies provide new opportunities for the potential future development of the CHM (Yang et al., 2015). In the last decade, significant developments have been achieved in epigenomics studies, focusing on the complete set of epigenetic modifications. Unlike the classic Mendelian inheritance, which focuses on mutations of the DNA sequence, the epigenetic modifications are reversible and heritable; they can modify the gene expression without altering the DNA sequence (Elhamamsy, 2017; Feinberg, 2018) (Figure 1). Thus, epigenetic modifications play an essential role in regulating gene expression and are crucial for various cellular processes (Wilkinson and Gozani, 2017).

**FIGURE 2:**
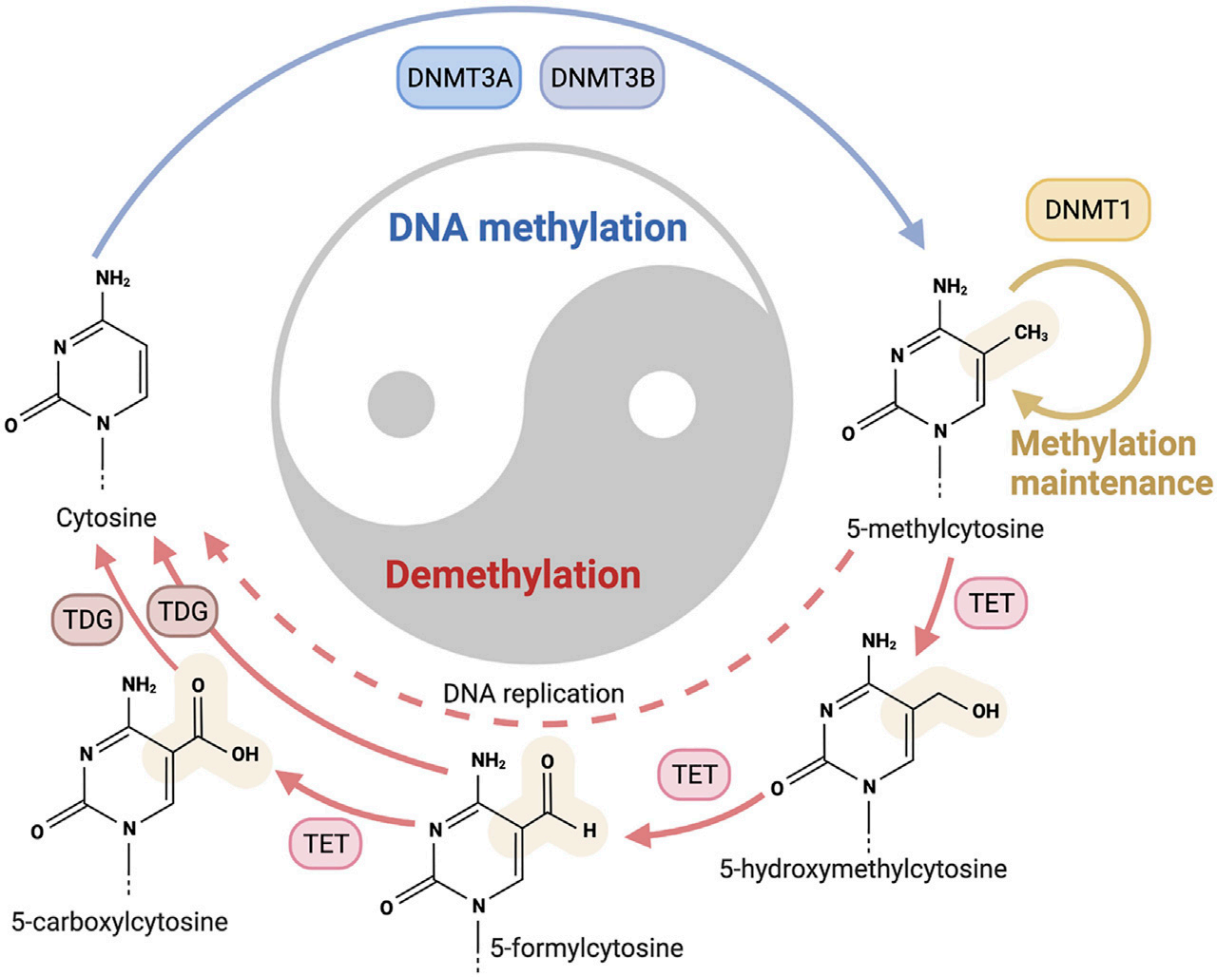
Processing of DNA methylation and demethylation, these two mechanisms may achieve a changeable balance. The formation and maintenance of DNA methylation need the catalysis of DNA methyltransferases (DNMTs) to produce 5-methyl-cytosine from cytosine (indicated with blue line). The demethylation process contains two pathways. The passive pathway refers to the dilution of the modified cytosines during DNA replications (indicated with red dashed lines). The active pathway of demethylation to reverse 5-methyl-cytosine to cytosine, enzymes of ten-eleven translocation (TET), and thymine DNA glycosylase (TDG) participate in this process (indicated with red lines).

DNA methylation is one of the most broadly studied and well-characterized epigenetic modifications (Nowacka-Zawisza and Wisnik, 2017; Draht et al., 2018; Islam et al., 2018). DNA methylation involves adding a methyl group to the 5-carbon position of the cytosine ring of DNA by DNA methyltransferases (Schubeler, 2015) (Figure 2). DNA methylation plays a crucial role in gene expression and regulation, and faulty methylation could induce devastating consequences, including various fatal human diseases, such as cancer, lupus, and cardiovascular diseases (Liu et al., 2017; Fernandez-Sanles et al., 2018; Imgenberg-Kreuz et al., 2018).

**FIGURE 3:**
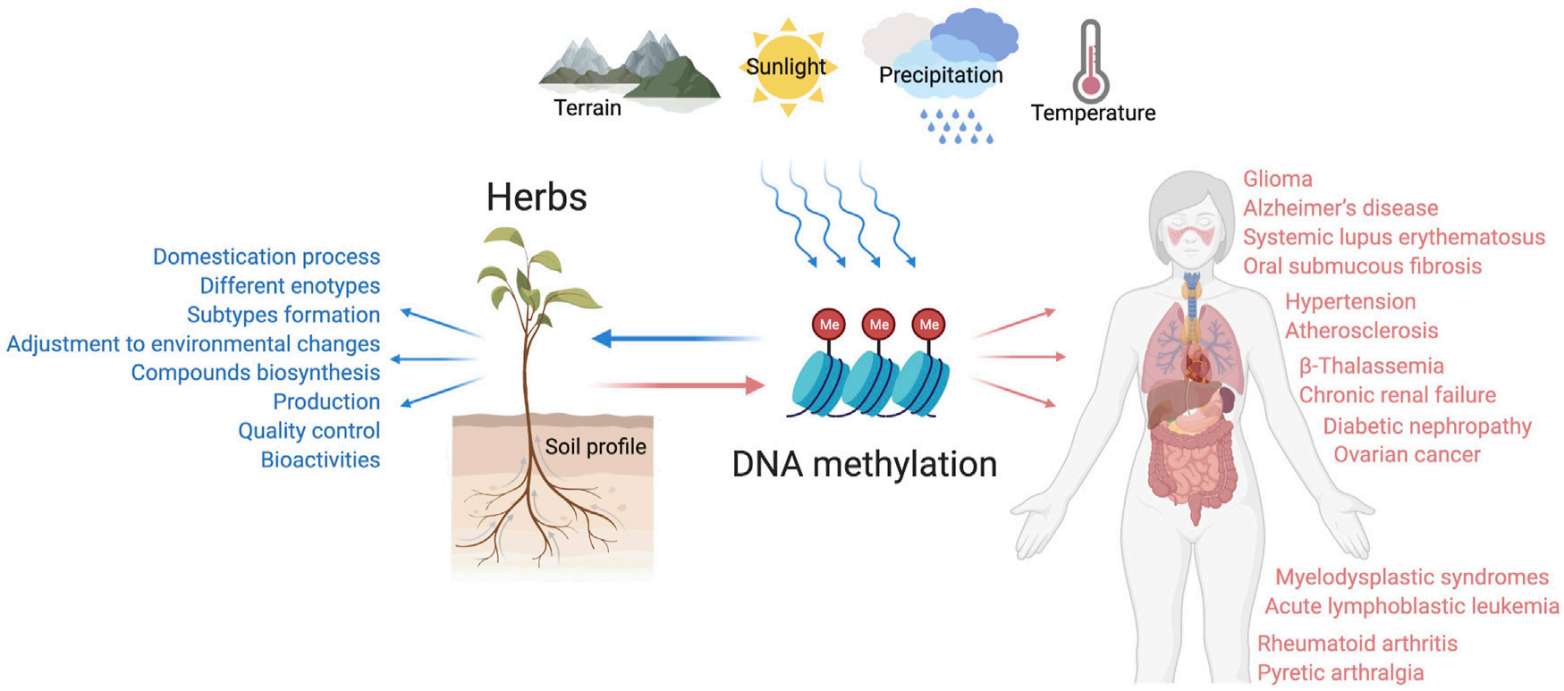
The pleiotropic role of DNA methylation in the growth and pharmacological activities of CHM botanical drugs. DNA methylation serves as a mediator mechanism for the medicinal plants to respond to the ever-changing environmental factors, which affect the domestication process, subtypes formation, compounds biosynthesis, and many other bioprocesses. DNA methylation is also indispensable for herbs to exert therapeutic functions related to multisystem diseases, such as atherosclerosis, hypertension, Alzheimer’s disease, ovarian cancer, rheumatoid arthritis, etc.

Multiple technologies have been applied in the research of CHM, and fruitful outcomes have been achieved (Wang et al., 2013; Gu and Pei, 2017). The recent development of DNA methylation technique sheds new light on the CHM research, and many related studies have also been conducted, which helped to provide a better understanding of some puzzling issues (Hsieh et al., 2011; Liu et al., 2016; Yao et al., 2018b) (Figure 3). In this review, the application of DNA methylation in CHM research will be presented, and specific attention will be placed on its clinical utility; attempts will be made to elucidate the research trends in this field to benefit future studies.

## Concept and Biofunctions of DNA Methylation

DNA methylation is a dynamic state which reflects the changeable balance achieved by DNA methyltransferase-induced methylation and DNA demethylase-induced demethylation (Figure 2). DNA methylation exerts comprehensive biofunctions in plants and mammals, and displays great significance in the medical field.

### Patterns of DNA Methylation and Demethylation

DNA methylation is a very common biological phenomenon that exists in most eukaryotic organisms including both animals and plants, although they have different manifestations. In mammals, DNA methylation mainly occurs in the context of CG to form a cytosine-guanine dinucleotide (CpG) site; while, in plants, there are more types of DNA methylation, it may occur in all cytosine sequence contexts of CG, CHG, and CHH (H = A, C, or T) (He et al., 2011).

In mammals, DNA methylation is catalyzed by DNA methyltransferases (DNMTs), i.e., DNMT1, DNMT3A, and DNMT3B. The *de novo* DNA methylation is mainly induced by DNMT3A and DNMT3B, and DNMT1 is crucial for the maintenance of DNA methylation (Figure 2). DNA methylation can be reversed by demethylation which contains passive and active pathways (Greenberg and Bourc’his, 2019). During DNA replication, DNA methylation may be diluted, and this process is the passive pathway of DNA demethylation. The active pathway of demethylation is catalyzed by enzymes of ten-eleven translocation (TET), and thymine DNA glycosylase (TDG) (Kohli and Zhang, 2013).

In plants, the *de novo* DNA methylation is mediated by three types of DNMTs: MET1, CMT3, and DRM2. The maintenance of CG methylation mainly relies on MET1 which is a homolog of DNMT1 in mammals, while the maintenance of CHG DNA methylation is catalyzed by CMT3. DRM2 induces *de novo* DNA methylation at all sequence contexts in plants through the RNA-directed DNA methylation (RdDM) pathway (He et al., 2011; Zhang et al., 2018). As for DNA demethylation in plants, the passive pathway is similar to mammals, whereas the active DNA demethylation pathway is distinct. Plants can directly remove the 5-methylcytosine (5-mC) base by 5-mC DNA glycosylases which is different from the way mammals initiate active DNA demethylation by the oxidation and deamination of 5-mC (Zhang et al., 2018).

### Biofunctions of DNA Methylation

DNA methylation plays essential roles in diverse biological processes involving the regulation of gene expression, transposon silencing, chromosome interactions, and trait inheritance (Luo et al., 2018). The most noticeable molecular function of DNA methylation is gene regulation. When DNA methylation occurs in the promoter region it usually inhibits gene transcription, for DNA methylation can impede the binding of transcription factors to their motifs, thus the transcription process can be hindered (Yin et al., 2017) (Figure 1). DNA methylation also participates in the process of heterochromatin formation via recruiting the chromatin remodelers and modifiers by DNMT proteins to chromatin (Greenberg and Bourc’his, 2019). Except for genes, the main targets of DNA methylation are transposable elements, and transposons may harm genome stability by relocating DNA transposons or inserting retrotransposons. CpG-rich promoters control the expression of active retrotransposons. DNA methylation can induce the silence of transposons and promote the irreversible genetic inactivation of transposable elements through mutagenic deamination, and thus support genome stability (Barau et al., 2016). DNA methylation can also reprogram the development of both mammals and plants, exert regulatory effects in the bioprocesses of early embryogenesis, X chromosome inactivation, genomic imprinting, stem cell differentiation (He et al., 2011; Schubeler, 2015; Zhang et al., 2018). Aberrant DNA methylation is shown to be involved in the occurrence and development of multiple diseases, a host of promising diagnostic and therapeutic approaches based on DNA methylation has been explored and partially applied in the clinical practice (Dor and Cedar, 2018; Papanicolau-Sengos and Aldape, 2021) (Figure 3). DNA methylation research also sheds new light on the field of CHM, providing a perspective to unveil the obscure mechanisms behind the profound CHM clinical experience.

## Dna Methylation in the Growth and Development of Medicinal Plants in Chinese Herbal Medicine

Planting and growth are the key basis for the curative effects of CHM herbs. DNA methylation is an effective mechanism that participates in multiple biological processes of the growth and development of medicinal plants, such as subtypes domestication, development, and the adjustment to dynamic environments (Table 1). Investigations of several selected CHM medicinal plants are presented below.

**TABLE 1:** DNA methylation in the growth and adaptation to ecological changes of CHM medicinal plants.

Medicinal plants	Activities	Main findings	References
*Panax ginseng* C. A. Mey.	DNA methylation	DNA methylation participates in the mechanism of domestication process of northern and southern cultivated ginseng subtypes	Li et al. (2017c)
*Salvia miltiorrhiza* Bge.	DNA methylation	DNA methylation is involved in medicinal plant development and bioactive compound biosynthesis, affects the secondary metabolism and stress response in *S. miltiorrhiza*	Li et al. (2018a)
*Pinellia ternata* (Thunb.) Breit.	DNA methylation and demethylation	Planted with shading induced 32.51% of the gene DNA methylation and 16.25% demethylation, and effectively promoted the yield	Shi et al. (2020)
*Viola elatior* Fries	DNA methylation	DNA methylation plays an important role in the rapid adjustment of plant populations to dynamic environmental conditions	Schulz et al. (2014)
*Helleborus foetidus* L.	DNA methylation	DNA methylation may influence phenotypes and ecological processes in wild plant populations	Alonso et al. (2014)
*Hydrilla verticillata* (L.f.) Royle	DNA methylation	Excess exposure to copper can affect the expressions of methyltransferases, furthermore, copper-induced reactive oxygen species may cause the DNA damage and result in DNA methylation in *H. verticillata*	Shi et al. (2017)

*Panax ginseng* C. A. Mey. (Araliaceae; Ginseng Radix et Rhizoma) originates in China and has been one of the most widely used herbal medicines globally, with a history of over a thousand years (Dai et al., 2017; Yao et al., 2018a). DNA methylation has been reported to be related to the domestication process and quality control of *P. ginseng*. There are several different subtypes of *P. ginseng*, and distinct phenotypic and genetic attributes could be identified among landraces. Through genome sequencing and phylogenetic analysis of wild and cultivated *P. ginseng* accessions, the northern cultivated *P. ginseng* and southern cultivated *P. ginseng* have undergone different selection pressures during the domestication process. Functional analyses revealed that DNA methylation is related to the different genes, which suggested that DNA methylation may participate in the domestication process of *P. ginseng* subtypes (Li MR. et al., 2017). The accumulation of ginsenosides is crucial for the quality control of *Panax quinquefolium* L. (Araliaceae; Panacis Quinquefolii Radix); the cold environment plays a decisive role in this process. It has been revealed that sufficient cold exposure duration in winter may induce sufficient DNA demethylation in tender leaves in early spring; this is closely correlated with the increased ginsenosides accumulation in the roots (Hao et al., 2020).

*Salvia miltiorrhiza* Bge. (Lamiaceae; Salviae Miltiorrhizae Radix et Rhizoma) is another frequently-used Chinese herbal medication (Yu et al., 2015; Zhang Y. et al., 2017). In plants, the epigenetic modification of cytosine DNA methylation was established and maintained by cytosine-5 DNA methyltransferases (C5-MTases). Through genome-wide analyses, eight putative SmC5-MTase genes were identified from the genome of *S. miltiorrhiza*. Moreover, the further transcript abundance analysis suggested the functional importance of SmC5-MTases in the secondary metabolism and stress response in *S. miltiorrhiza*. This research provided helpful information to reveal the role of DNA methylation in medicinal plant development and bioactive compound biosynthesis (Li J. et al., 2018).

*Pinellia ternata* (Thunb.) Breit. (Araceae; Pinelliae Rhizoma) is a typical Chinese herbal medicine with great demand in the Chinese market. It was found in planting that shade can effectively increase its yield. To explore its reasons, *P. ternata* grew under natural light, and 90% shading was selected as the control and experimental groups for genomic DNA methylation analysis using methylate sensitive amplification polymorphism (MSAP). The results showed that compared to the natural light group, shading induced 32.51% of the gene DNA methylation and 16.25% demethylation, which revealed that DNA methylation variations might participate in the mechanism of increased production of *P. ternata* under shading conditions (Shi et al., 2020).

Epigenetic modifications work as compensations for the relatively slow response of genetic adaptations. Through surveying the populations of *Viola elatior* Fries (Violaceae) from two adjacent habitat types, investigated by amplified fragment length polymorphisms and methylation-sensitive amplification polymorphisms analyses, researchers found that DNA methylation may have been playing an essential role in the rapid adjustment of plant populations to dynamic environmental conditions (Schulz et al., 2014). Furthermore, DNA methylation may also influence phenotypes and ecological processes in wild plant populations, and epigenetic variation can also predict the regional and local intraspecific functional diversity. Studies investigating the natural variation in global DNA cytosine methylation and its phenotypic correlations in the perennial herb *Helleborus foetidus* L. (Ranunculaceae) have confirmed this result (Alonso et al., 2014; Herrera et al., 2014; Medrano et al., 2014).

The environment is a significant factor that influences DNA methylation. Excess exposure to copper can induce DNA methylation changes in *Hydrilla verticillata* (L.f.) Royle (Hydrocharitaceae) in two different ways. On the one hand, the excess copper stress can affect the expressions of methyltransferases and then regulate the DNA methylation patterns; on the other hand, the copper-induced reactive oxygen species may cause DNA damage and then result in DNA methylation (Shi et al., 2017).

DNA methylation in botanical studies has revealed that this mechanism can explain how different environmental factors affect plants’ growth and adaptation to ecological changes. The botanical growth at different environmental conditions will likely affect the quality control of CHMs and subsequently affect their clinical therapeutic outcome.

## DNA Methylation in the Pharmacological Mechanisms of Chinese Herbal Medicine

Due to the complexity of ingredients and the uncertainty of the pharmacological mechanisms, the term “black box” is usually to be applied to depict this dilemma of CHM. However, with the rapid advance of scientific research, the condition has been greatly improved, DNA methylation and other epigenetic approaches contribute markedly to revealing the pharmacological mechanisms of CHM formulae, botanical drugs, and herbal-derived compounds.

### Chinese Herbal Medicine Formulae

Many classical CHM formulae with a long history have been widely used in various clinical departments, to uncover their pharmacological mechanisms through DNA methylation studies may also contribute the modern clinical practice. Estrogen receptor α (*ERα*) can protect against atherosclerosis (AS), the hypermethylation can reduce the expression of the gene ERα. Classical CHM formula Liuwei Dihuang Decoction (LDD) has been reported to enhance *ERα* expression to prevent AS. An *in vitro* study has shown that LDD protects human umbilical vein endothelial cells (HUVECs) from apoptosis induced by Hcy. Treatment with LDD significantly increased the expression of *ERα* and reduced the rate of *ERα* gene methylation by inhibiting the expression of DNMT1. The level of primary methyl donor SAM and the SAM/SAH ratio were both reduced by LDD. ApoE^−/-^ mice were ovariectomized to establish a model of postmenopausal AS. The results displayed that LDD effectively prevented plaque formation and reduced the concentration of Hcy; furthermore, LDD upregulated *ERα* expression and inhibition of DNMT1 expression in atherosclerotic mice, validating the similar epigenetic regulation effects in HUVECs experiments (Chen et al., 2020).

Another CHM formula also displayed beneficial effects on AS via an epigenetic modification to regulate the expression of *ERα*. San-Huang-Xie-Xin-Tang (SHXXT) showed better effects compared with Statin to restore microRNA-152, decrease DNMT1, down-regulate the methylation level of *ERα*, and finally increase *ERα* expression in both cellular and animal studies (Wang et al., 2012).

Wutou Decoction (WTD) is a classical formula of CHM; it has been wildly applied in treating rheumatoid arthritis for many years (Zhang et al., 2015; Guo et al., 2017). Collagen-induced arthritis (CIA) rat model was established to explore the effects of WTD on the epigenetic modifications of rheumatoid arthritis. The data showed that the DNA methylation level was significantly down-regulated in group WTD than in group CIA, and H3 acetylation of peripheral blood mononuclear cells (PBMCs) was overexpressed in WTD compared with CIA. This result indicated that DNA methylation might play a significant role in WTD treatment of rheumatoid arthritis. WTD modulates DNA methylation and histone modifications, functioning as an anti-inflammatory potential (Liu et al., 2016).

Baihu Guizhi Decoction (BGD) has specific curative effects on pyretic arthralgia (PA), according to CHM practice. To investigate the characteristic methylation genes of the PA model showed that the whole-genome CpG island in the PA group was kept in a lower methylation state than the standard group. The intervention of BGD significantly ameliorated the foot swelling and pathological injury in PA models, decreased the levels of IL-1β, TNF-α, EGF, IL-17, and other inflammatory factors. It also inhibited the mRNA expression levels of down-regulated methylation genes *Ahcy* and *Rpl3* and promoted the mRNA expression levels of upregulated methylation gene *Agxt*. The results indicated that BGD achieved the therapeutic effects on PA by adjusting characteristic genes’ methylation levels (Chen et al., 2017).

Xuefu Zhuyu Decoction (XZD) is a widely used CHM formula in treating multiple circulation system diseases. A group of 49 patients diagnosed with clopidogrel resistance (CR) were randomly divided into control and treatment groups, and the DNA methylation level of gene *P2Y12* was inspected with a bisulfite pyrosequencing assay. Results indicated that XZD improved CR, and the *P2Y12* polymorphisms and their methylation level influenced the drug effect. Patients with lower methylation levels of CpGI were more likely to be TT genotype in rs2046934 and received substantial benefits from XZD (Yu et al., 2019).

Besides the classical CHM formulae, DNA methylation research has also been conducted in pharmacological investigations into the modern composed formulae. Qian Yang Yu Yin Granule (QYYYG) is a Chinese herbal formulation that has been used to treat hypertensive nephropathy for decades in China. The proliferation of HEK293T cells induced by Angiotensin II was chosen to observe the epigenetic mechanism of QYYYG on renal damage. It was found that QYYYG inhibited HEK293T cells’ proliferation, down-regulated the expression of Nicotinamide N-Methyltransferase (NNMT), S-adenosylhomocysteine (SAH), acetyl-cortactin, and DNA methylation. The results revealed that QYYYG alleviated renal impairment in spontaneously hypertensive rats and inhibited the proliferation of HEK293T cells through the pathways linked to DNA methylation and other epigenetic mechanisms (Zhang et al., 2020).

Refractory cytopenia with multilineage dysplasia (RCMD) is one of the most common types of myelodysplastic syndromes (MDS) (Iwafuchi and Ito, 2018). Qinghuang Powder (QHP) has been applied to treat MDS for over 30 years and has proven effective in the CHM practice. To elucidate the mechanism of QHP in treating RCMD, clinical trials were conducted. Patients with MDS-RCMD were treated with QHP, and then bone marrow samples were obtained for further DNA methylation analysis. It was revealed that, through significant genome-wide DNA demethylation, QHP might achieve the hematologic improvement of MDS patients, suggesting this CHM formula may provide another choice for MDS treatment (Sun et al., 2012; Zhou et al., 2018).

β-Thalassemia, common in many parts of the world, is a hereditary disease resulting from red blood pigment hemoglobin (Sirisena et al., 2018). Yisui Shengxue Granules (YSSXG), a Chinese herbal medication, has been used as an alternative therapy for this disease. A double-blind, randomized, placebo-controlled trial was designed to examine the therapeutic effects of YSSXG and its influence on global DNA methylation. Apparent increases in hemoglobin, red blood cells, and fetal hemoglobin counts were observed in the treatment group. Moreover, DNA methylation examination screened 24 hypomethylated and 3,685 hypermethylated genes after the intervention. Further gene functional analysis suggested that JAK3 and MAPK10 were critical genes in the bone marrow and the lymphatic system; JAK3 was likely to be related to hematopoietic cytokines in the process of early hematopoiesis (Cheng et al., 2018).

Systemic lupus erythematosus (SLE) is a refractory autoimmune disease in which the body’s immune system mistakenly attacks healthy tissue in many parts of the body. It shows a high incidence rate in women of child-bearing age (De Risi-Pugliese et al., 2018; Di Battista et al., 2018). Clinical observation showed that Lang-Chuang-Ding Decoction (LCD), a CHM formula, could significantly reduce SLE disease activity index (SLEDAI); a further study aimed to investigate its effect on the DNA methylation of CD70 gene of patients with SLE. PBMCs isolated from female patients with SLE or healthy donors were cultured and treated with LCD medicated serum or normal serum. The mRNA and DNA methylation expressions of the CD70 gene were detected. The result showed that, by promoting the DNA methylation of CD70 gene promoter, LCD could inhibit the expression of gene CD70, which unveiled the potential mechanism of LCD to treat SLE (Sun et al., 2018). Furthermore, the Jieduquyuziyin Prescription (JP) is also an effective CHM formula for SLE. *In vitro* experiments with Jurkat T cells demonstrated that JP upregulated the levels of DNA methylation, MeCP2 mRNA, and protein as effectively as prednisone acetate, and thus may activate the MeCP2 gene by increasing the methylation level, thereby inhibiting the pathogenesis of SLE (Li R. et al., 2018).

Uremic Clearance Granule (UCG) has been used to treat chronic renal failure (CRF) in clinical practice for many years. Adenine-induced CRF was established in Wister rats to explore the mechanism of the therapeutic effects. After treatment with UCG, the kidney function of CRF rats was recovered. *Tgf-β1* promoter was demethylated at some loci in the pathological control group. UCG could also recover the methylation level of gene *Tgf-β1* and then regulate the transcription and translation of the gene, indicating that the DNA methylation may participate in the molecular mechanism of the therapeutic effect (Miao et al., 2010).

### Chinese Herbal Medicine Botanical Drugs and Compounds

With multiple pharmacological effects, Ginseng Radix et Rhizoma is one of the world’s most commonly used natural products (Wan et al., 2017; Wang et al., 2018). Ginsenoside Rg3, a bioactive constituent isolated from Ginseng Radix et Rhizoma, has shown antitumorigenic activities (Wu et al., 2018). Moreover, a further study indicated that DNA methylation was involved in this bioprocess (Teng et al., 2017). It was demonstrated that ginsenoside Rg3 could reduce global genomic DNA methylation and alter cytosine methylation of the promoter regions of some related genes. This epigenetic modification was associated with increased cell growth inhibition in the HepG2 human hepatocarcinoma cell line (Teng et al., 2017). Ginsenoside Rg3 has also been shown to increase the expression of tumor suppressor von Hippel-Lindau (*VHL*) gene in ovarian cancer cells via suppressing DNMT3A expression and thus downregulating the DNA methylation level of the *VHL* promoter (Wang et al., 2021). The Warburg effect is associated with increased glycolysis and rapid energy production; it has been remarked as one of the metabolic hallmarks of cancer. Ginsenoside Rg3 could down-regulate DNA methyltransferase DNMT3A and decrease the methylation in the promoter region of miR-519a-5p precursor gene, thus upregulating miR-519a-5p to inhibit the HIF-1α-stimulated Warburg effect in ovarian cancer (Lu et al., 2020). On MCF-7 breast cancer cells, ginsenoside ginsenoside Rh2 intervention down-regulated the methylation level of *LINE1*, which is a global methylation marker; ginsenoside Rh2 also induced genome-wide DNA methylation changes mapped to genes associated with immune response and tumorigenesis, thereby exerting anti-tumor effects (Lee et al., 2018).

Salviae Miltiorrhizae Radix et Rhizoma has been widely used in CHM practice for centuries (Rongrong et al., 2016; Zhang Y. et al., 2017). Tanshinones, isolated from *S. miltiorrhiza*, is a class of abietane diterpene compounds that contain three major types, those are cryptotanshinone (CT), tanshinone IIA (T2A), and tanshinone I (T1). Oral submucous fibrosis (OSF) induced by chewing the areca nut has been considered a precancerous lesion. Tanshinone intervention could suppress arecoline-induced epithelial-mesenchymal transition in oral submucous fibrosis and reverse the hypermethylation of *TP53* promoter induced by arecoline. Through down-regulating the DNA methylation level of the *TP53* promoter, tanshinone recovered the expression of p53 and reactivated the p53 pathway. Thus, tanshinone played an inhibitory role in the progression of OSF (Zheng et al., 2018).

Tanshinones, especially the T1, showed a growth inhibition effect on breast cancer cells in a dose-dependent manner. T1 also significantly reduced acetylation levels of histone H3 associated with the Aurora-A gene, implying tanshinones could inhibit the growth of breast cancer cells through the epigenetic modification of Aurora-A expression (Gong et al., 2012). T2A could induce demethylation of *Nrf2* and activate the Nrf2 antioxidative stress signaling pathway, potentially contributing to the attenuation of JB6 cellular transformation and anticancer effects (Wang et al., 2014). T2A also demonstrated a potential preventive effect on the high glucose-induced diabetic nephropathy cellular model, and DNA methylation may play a role in this process (Li W. et al., 2019).

Schisandrin B, derived from Schisandrae Chinensis Fructus, is an active agent who can exert various pharmacological activities. It has been proved to have significant protective effects against Alzheimer’s disease (AD) (Hu et al., 2018). Cell experiments revealed that schisandrin B could significantly inhibit the Aβ_1-42_ -induced damages in the SH-SY5Y neuronal cell line *via* the regulation of DNMT3A and DNMT1 mRNA expression then influence the DNA methylation level (Zhang M. et al., 2017). Further validation, such as *in vivo* experiments or clinical trials, is still needed to make this discovery go even more profound.

Curcumin is an active component of Curcumae Longae Rhizoma. Previous studies indicate curcumin possesses several biological activities beneficial to cancer treatment and prevention (Bano et al., 2018; Khorsandi et al., 2018; Nagaraju et al., 2018). Examined in human glioblastoma cells, DNA methylation may play a potential role in the process of curcumin to upregulate the *RANK* gene expression at both mRNA and protein levels. Curcumin induced a decreased methylation level of the *RANK* gene promoter, then reactivated gene *RANK*; thus, the alleviating effect to glioma could be achieved (Wu et al., 2013).

Triptolide is a component derived from the Chinese herb *Tripterygium wilfordii* Hook. f. and has potent immunosuppressive and anti-inflammatory effects. Triptolide and tripchlorolide exert beneficial effects on synapses in Alzheimer’s disease mice, and the underlying mechanism may be related to DNA methylation, for the compounds can inhibit the DNA methylation of *Nlgn1* promoter in the hippocampus, and thus increase the expression of gene *Nlgn1* (Lu et al., 2019)*.* Evaluated *in vitro*, triptolide was shown to have the effect of inhibiting the growth and proliferation of acute lymphoblastic leukemia Jurkat cell line in dose-and time-dependent manners. Triptolide can also reverse the hypermethylation of antioncogene *APC* and upregulate the *APC* expression, which may provide a potential mechanism of triptolide’s protective effects (Wu et al., 2010).

As a monotherapy or adjunct therapy, many CHM formulae and bioactive compounds have displayed beneficial pharmacological activities in treating various modern diseases (Table 2, 3). Elucidating the mechanism of these activities is essential for the safety and effectiveness of CHM clinical application. However, the complexity of ingredients, the multi-targeting treatment strategy, and the guidance of the holistic concept all increase the ambiguity of CHM. The recent development of epigenetics, especially DNA methylation research, facilitates uncovering the hidden secrets of CHM. As an epigenetic modification, DNA methylation serves as an intermediate factor playing an essential role in the mechanism of CHM to exert therapeutic effects (Figure 3).

**TABLE 2:** DNA methylation participates in multiple pharmacological effects of CHM formulae.

CHM formulae	Compositions	Research models	Activities	Pharmacological effects	References
Wutou Decoction (WTD)	Ephedrae Herbal 9 g, Paeoniae Rubra Radix 4.5 g, Paeoniae Alba Radix 4.5 g, Astragali Radix 9 g, Glycyrrhizae Radix et Rhizoma 9 g, Aconiti Radix 6 g	Collagen-induced arthritis rat model	Down-regulated DNA methylation	DNA methylation may play a significant role in WTD treatment of rheumatoid arthritis	Liu et al. (2016)
Qinghuang Powder (QHP)	Indigo Naturalis 0.24 g, Realgar 0.16 g (capsule)	Clinical trial, 25 patients with myelodysplastic syndromes (MDS) were included	Genome-wide demethylation	QHP achieves the hematologic improvement of patients with MDS through a significant genome-wide DNA demethylation	Sun et al. (2012)
Yisui Shengxue Granules (YSSXG)	Corni Fructus, Polygoni Multiflori Radix, Rehmanniae Radix, Astragali Radix, Codonopsis Radix, Angelicae Sinensis Radix, Psoraleae Fructus, Asini Corii Colla, Spatholobi Caulis, Trionycis Carapax and Amomi Fructus (12 g/bag)	Randomized double-blinded, placebo- controlled clinical trial, 40 patients with β-Thalassemia were included	Induced 24 hypomethylated and 3,685 hypermethylated genes	JAK3 and MAPK10 are two key genes in bone marrow and the lymphatic system which are involved in the treatment of β-Thalassemia with YSSXG	Cheng et al. (2018)
Lang-Chuang-Ding Decoction (LCD)	Rehmanniae Radix 15 g, Trionycis Carapax 24 g, Cimicifugae Rhizoma 9 g, Hedyotis Herba 12 g, Artemisiae Annuae Herba 12 g, Centellae Herba 15 g, Paeoniae Rubra Radix 12 g, Coicis Semen 15 g, Citri Sarcodactylis Fructus 9 g, Glycyrrhizae Radix et Rhizoma 6 g	Peripheral blood mononuclear cells isolated from female patients with systemic lupus erythematosus (SLE)	Promoting the DNA methylation of CD70 gene promoter	LCD could significantly reduce the disease activity index of SLE, and inhibit the expression of gene CD70 *via* promoting DNA methylation	Sun et al. (2018)
Jieduquyuziyin Prescription (JP)	Astragali Radix, Testudinis Carapax et Plastrum, Artemisiae Argyi Folium, Hedyotis Herba, Centellae Herba, Peoniae Radix Rubra, Moutan Cortex, Citri Sarcodactylis Fructus, Cimicifugae Rhizoma, Glycyrrhizae Radix et Rhizoma	Jurkat T cells	Up-regulated the DNA methylation level of *MeCP2*	JP inhibites the pathogenesis of SLE through upregulating the levels of MeCP2 gene promoter region methylation in Jurkat cells	Li et al. (2018b)
Qian Yang Yu Yin Granule (QYYYG)	Polygoni Multiflori Radix, Bidentis Bipinnatae Herba, Corni Fructus, Scrophulariae Radix, Alismatis Rhizoma, Cyathulae Radix	HEK293T cells	Down-regulated DNA methylation	QYYYG alleviates renal impairment in spontaneously hypertensive rats, and down-regulated the expression levels of Nicotinamide N-methyltransferase, S-adenosylhomocysteine, and DNA methylation	Zhang et al. (2020)
Xuefu Zhuyu Decoction (XZD)	Persicae Semen, Carthami Flos, Aurantii Fructus, Platycodonis Radix, Cyathulae Radix, Bupleuri Radix, Rehmanniae Radix, Angelicae Sinensis Radix, Chuanxiong Rhizoma, Paeoniae Radix Rubra, Glycyrrhizae Radix et Rhizoma	Clinical trial, 49 patients with clopidogrel resistance (CR) were included	Hypomethylation of gene *P2Y12*	CR patients with lower methylation levels are more likely to be TT carriers in rs2046934 may influnce the clinical effect of XZD	Yu et al. (2019)
Liuwei Dihuang Decoction (LDD)	Rehmanniae Radix, Corni Fructus, Dioscoreae Rhizoma, Alismatis Rhizoma, Moutan Cortex, Poria	Human umbilical vein endothelial cells	Down-regulated DNA methylation of gene *ERα*	LDD displayes protective effects on postmenopausal atherosclerosis mice *in vivo*, and human umbilical vein endothelial cells treated with Hcy *in vitro*, increases *ERα* expression *via* inhibiting DNMT1-dependent *ERα* methylation	Chen et al. (2020)
San-Huang-Xie-Xin-Tang (SHXXT)	Coptidis Rhizoma, Scutellariae Radix, Rhei Radix et Rhizoma	Human aortic smooth muscle cells	Down-regulated DNA methylation of gene *ERα*	This formula exertes beneficial effects on atherosclerosis through restoring microRNA-152, decreasing DNMT1, down-regulating the methylation level of *ERα*	Wang et al. (2012)
Uremic Clearance Granule (UCG)	Rhei Radix et Rhizoma, Astragali Radix, Mori Cortex, Sophorae Flavescentis Radix, Atractylodis Macrocephalae Rhizoma, Poria, Paeoniae Radix Alba, Polygoni Multiflori Radix Praeparata, Salviae Miltiorrhizae Radix et Rhizoma, Plantaginis Herba, *et al*	Adenine induced chronic renal failure (CRF) rat model	Up-regulated DNA methylation of gene *Tgf-β1*	UCG could revover the kidney function of CRF rats, and restore the DNA methylation of gene *Tgf-β1* to normal level	Miao et al. (2010)
Baihu Guizhi Decoction (BGD)	Gypsum Fibrosum 60 g, Anemarrhenae Rhizoma 15 g, Glycyrrhizae Radix et Rhizoma 5 g, Oryza Sativa Semen 30 g, Cinnamomi Ramulus 10 g	Pyretic arthralgia rat model was induced by plantar injection of CFA	Adjusted the DNA methylation levels of *Ahcy*, *Rpl3*, and *Agxt*	BGD achieves the therapeutic effects on pyretic arthralgia through adjusting the methylation levels of characteristic genes	Chen et al. (2017)

**TABLE 3:** DNA methylation participates in multiple pharmacological effects of botanical drugs and compounds.

Botanical drugs	Compounds	Research models	Activities	Pharmacological effects	References
Ginseng Radix et Rhizoma	Ginsenoside Rg3	Human hepatocarcinoma cells	Reduced global genomic DNA methylation and some related genes	Rg3 shows antitumorigenic activities which were related to the regulation of DNA methylation	Teng et al. (2017)
Ginseng Radix et Rhizoma	Ginsenoside Rg3	Ovarian cancer cells	Down-regulated the DNA methylation of *VHL* promoter	Rg3 upregulates *VHL* expression in ovarian cancer cells by suppressing DNMT3A-mediated DNA methylation	Wang et al. (2021)
Ginseng Radix et Rhizoma	Ginsenoside Rg3	Ovarian cancer cells	Down-regulated the DNA methylation	Rg3 decreases the DNMT3A-mediated DNA methylation thus upregulates miR-519a-5p to inhibit the HIF-1α-stimulated Warburg effect in ovarian cancer	Lu et al. (2020)
Ginseng Radix et Rhizoma	Ginsenoside Rh2	Breast cancer cells	Down-regulated the DNA methylation of *LINE1* and regulate genome-wide methylation profile	Rh2 induces genome-wide DNA methylation changes mapped to genes associated with immune response and tumorigenesis, thereby exerting anti-tumor effects	Lee et al. (2018)
Salviae Miltiorrhizae Radix et Rhizoma	Tanshinone	Human oral mucosal fibroblasts	Down-regulated the DNA methylation level of *TP53* promoter	Tanshinone suppresses arecoline-induced epithelial-mesenchymal transition in oral submucous fibrosis by reversing the hypermethylation of TP53 and thus reactivating the p53 pathway	Zheng et al. (2018)
Salviae Miltiorrhizae Radix et Rhizoma	Tanshinone IIA (T2A)	Mouse skin epidermal JB6 cells	Down-regulated the DNA methylation of *Nrf2*	T2A could induce demethylation of *Nrf2* and activate the Nrf2 signaling pathway, potentially contribute to the attenuation of JB6 cellular transformation and anticancer effects	Wang et al. (2014)
Salviae Miltiorrhizae Radix et Rhizoma	Tanshinone IIA (T2A)	Mouse kidney mesangial mes13 cells	RNA expression changes were correlated with the differences in CpG methylation	DNA methylation may affect the potential preventive effect of T2A on diabetic nephropathy	Li et al. (2019b)
Schisandrae Chinensis Fructus	Schisandrin B	SH-SY5Y neuronal cells	DNA methylation	Schisandrin B is shown to have protective effects against Alzheimer’s disease in cell model via regulating DNMT3A and DNMT1 mRNA expression then influence the DNA methylation level	Zhang et al. (2017a)
Curcumae Longae Rhizoma	Curcumin	Glioblastoma cells	Down-regulated the DNA methylation of *RANK* gene promoter	Curcumin may achieve the alleviating effect to glioma by inducing decreased methylation and then reactivated gene *RANK*	Wu et al. (2013)
*Tripterygium wilfordii* Hook. f.	Triptolide	Acute lymphoblastic leukemia Jurkat cells	Down-regulated the DNA methylation of *APC*	Triptolide can reverse the hypermethylation of antioncogene *APC*, and inhibit the growth and proliferation of acute lymphoblastic leukemia Jurkat cell line	Wu et al. (2010)
*Tripterygium wilfordii* Hook. f.	Triptolide and tripchlorolide	APP/PS1 transgenic Alzheimer’s disease (AD) mice model	Down-regulated the DNA methylation of *Nlgn1* gene promoter	Triptolide and tripchlorolide may have protective effects on synapses in AD mice by inhibiting the DNA methylation of *Nlgn1* promoter in the hippocampus	Lu et al. (2019)

## DNA Methylation in Constitution Theory and Other Issues of Chinese Herbal Medicine

DNA methylation has not only been applied in pharmaceutical research but has also been demonstrated to be beneficial in elucidating basic theoretical research in CHM, for instance, the body constitution theory, syndrome differentiation, etc.

CHM uses a unique theoretical and practical system to diagnose and treat diseases. Syndrome differentiation is one of the primary approaches used in clinical practice and CHM higher education (Hua et al., 2017; Zhao, 2017). Based on the clinical information collected through the four diagnostic methods of observation, listening/smelling, inquiry, and palpation examination, the diagnosis of disease and syndrome can be drawn. However, if combined with constitution differentiation, the diagnosis could be more accurate. Constitution differentiation will also reveal more comprehensive information about the background of the disease (Xu et al., 2017), it enhances not only the disease treatment but also the disease prevention in CHM. Body constitution theory originates in the profound CHM clinical practice, which has been widely used in China and abroad and proved to be reliable and fruitful (Li L. et al., 2019). Constitution in CHM is the study of the intrinsic features of the human body, including congenital and acquired ones (Wang et al., 2011). There are nine basic constitution types in CHM, i.e., balanced constitution, qi-deficiency constitution, yang-deficiency constitution, yin-deficiency constitution, phlegm-dampness constitution, damp-heat constitution, blood stasis constitution, qi stagnation constitution, and inherited special constitution. Different constitution types have different characteristics and different predispositions. Therefore, based on the constitution identification, corresponding high-risk diseases can be predicted, creating the possibility of early prevention (Li M. et al., 2017; Ma et al., 2017; Liu et al., 2018).

Among the nine constitution types, the balanced constitution is thought to be the healthiest type. Phlegm-dampness Constitution (PC) is one of the most studied constitution types which predisposes individuals to complex metabolic disorders (Wang et al., 2013; Li L. et al., 2017). The theory of PC can effectively promote early prediction and prevention of metabolic diseases. It also provides a new perspective on the increasing prevalence of metabolic diseases and the related complications, which have already caused heavy medical and financial burdens in the global society (Wang et al., 2013). As a reversible epigenetic modification, DNA methylation can be used to explore the influence of postnatal factors on body constitution. Our group previously has applied the Infinium HumanMethylation450 BeadChip technique to explore the DNA methylation profiles of PC; through the DNA methylation research, the molecular mechanism of PC has been partially revealed (Yao et al., 2018b). DNA samples were extracted from peripheral blood mononuclear cells of PC and balanced constitution volunteers; the balanced constitution was set to be the control group. A genome-wide DNA methylation analysis was conducted, which indicated a genome-scale hyper-methylation pattern in PC and located 288 differentially methylated probes (DMPs). Based on these DMPs, 256 genes were mapped; some of these genes were metabolic-related and could be enriched in multiple metabolic gene pathways (Yao et al., 2018b). This research indicated PC has a metabolic disorder feature even in healthy individuals, thus, DNA methylation may play a potential role in this pathological mechanism. The study data should benefit the understanding of PC and explain why PC is prone to multiple metabolic diseases (Yao et al., 2018b; Tobi et al., 2018).

Syndrome differentiation is an essential theory in CHM diagnostics; however, the lack of biological basis limits its application and development in the context of modern science to a certain extent. The emerging DNA methylation research provides a new potential resolution to this issue. A study combined genome-wide DNA methylation and serum cytokine profiles was conducted to search the molecular bases of CHM syndrome classification in patients with chronic hepatitis B. As the results indicated that the hypomethylation of CpG loci in 5’ UTR of *HLA-F* may up-regulate *HLA-F* expression, furthermore, this parameter was significantly correlated with the cytokine levels of IL-12, MIP-1α, and MIP-1β, they may serve as a diagnostic marker for the syndrome differentiation of chronic hepatitis B (Hu et al., 2021). A similar approach was applied to explore the biomarker for syndrome differentiation in difficult-to-control asthma. Patients with hot syndrome showed hypermethylation at probe cg10791966 in gene *ALDH3A1* with corresponding lower mRNA expression, implying that *ALDH3A1* might be a potential biomarker for the syndrome differentiation of difficult-to-control asthma (Song et al., 2020). Besides the diagnostic application, aberrant DNA methylation can also serve as potential targets for CHM interventions, which may help to develop a novel clinical mode (Shen et al., 2021).

## Discussion and Conclusion

Chinese medicine, including CHM and acupuncture, has been attracting more attention due to its profitable clinical performance. With the doctrine of holism as a core concept, CHM can address the limitations of western medicine’s “one target, one drug” strategy. With profound clinical experience, CHM provides effective treatment solutions for many modern refractory diseases. However, barriers still hinder further exploration of CHM, as many mechanisms and hidden pearls of wisdom still need to be unveiled. As outlined above, epigenetic research, especially DNA methylation, presents an opportunity to address these challenges.

DNA methylation research is intensely beneficial and meaningful to CHM. DNA methylation, a dynamic changing and reversible process, has the features of heritability and variability. It promotes examining how herb growth and development is affected by environmental factors which may affect the quality control and efficacy of CHM herbs. DNA methylation also illuminates the pharmaceutical mechanisms of CHM formulae and compounds, which is meaningful in facilitating the clinical application of CHM. DNA methylation provides scientific validation and proves the clinical feasibility of CHM body constitution theory, some specific DNA methylation loci can serve as potential biomarkers to realize the diagnostic of CHM syndromes and constitutions at a molecular level. In addition, DNA methylation is widely distributed in the human body and not limited to a particular organ or system. CHM is a holistic medical system, and herbal medications work in a systematic and multi-targeted way. From this point of view, methylation may help to disclose the molecular mechanism and locate the potential drug targetsof CHM, which would be extremely significant for clinical practice.

The newly developed field of DNA methylation is up-and-coming for addressing many complicated issues of CHM. It could not only promote clinical treatment but, more importantly, also facilitate disease prevention. At present, investigators are concentrating on the epigenetic research of the CHM constitution, which aims to elucidate the molecular basis of different body constitution types. The obtained results are expected to reveal why different constitution types make individuals susceptible to various medical conditions. Epigenetic research of body constitution will possibly lay the foundation for disease prediction and prevention, which is vital to alleviate the heavy health care burden in modern society.

Currently, the field of epigenetic research in CHM is in its infancy. Although some inspiring studies have been conducted, the number of related publications is still somewhat limited. By reviewing the documents that we retrieved, some publications were not well-founded enough to achieve a solid conclusion, so further validating experiments are needed. More innovative and specific scientific approaches, more systematic experimental design, and practical techniques are required to improve the quality of the research of this domain. Furthermore, more in-depth integration is needed in future studies between CHM and epigenetics to apply CHM theory and clinical practice better.
